# Silent invaders: the role of MPs on epithelium inflammation and damage in airway diseases

**DOI:** 10.3389/falgy.2026.1758940

**Published:** 2026-02-16

**Authors:** Benedetta Bondi, Stefania Nicola, Federico Di Marco, Sara Chiappori, Luisa Brussino, Laura De Ferrari, Anna Maria Riccio, Fulvio Braido, Diego Bagnasco

**Affiliations:** 1 Respiratory and Allergic Clinic, IRCSS Azienda Ospedaliera Metropolitana, Genoa, Italy; 2 Department of Internal Medicine (DIMI), University of Genoa, Genoa, Italy; 3 Department of Medical Science, SCDU Immunology and Allergy - AO Ordine Mauriziano, Turin, Italy

**Keywords:** airways, asthma, epithelium, inflammation, microplastics

## Abstract

Microplastics (MPs) and Nanoplastics (NPs) have emerged as pervasive environmental contaminants with growing implications for respiratory health. Increasing evidence demonstrates that inhaled MPs can deposit throughout the airways, interact with epithelial surfaces, and trigger a cascade of inflammatory, oxidative, and structural alterations that may contribute to the onset or progression of airway diseases. Their pathogenicity is influenced by physicochemical properties, including size, shape, density, and surface charge, which determine their aerodynamic behavior, epithelial penetration, and cellular uptake. Once deposited, MPs are associated with epithelial stress responses, including oxidative stress, activation of inflammatory signaling pathways, and alterations in junction-related proteins, which may impair mucociliary function. Smaller particles and NPs are internalized through endocytosis, leading to mitochondrial dysfunction, reactive oxygen species (ROS) generation, and activation of key inflammatory pathways such as NF-κB, PI3K/Akt/mTOR, and Wnt/β-catenin. These mechanisms promote cytokine release, epithelial–mesenchymal transition, and dysregulated repair responses. Experimental and clinical evidence indicate that MPs exacerbate epithelial fragility in asthma and COPD by amplifying oxidative stress, enhancing barrier dysfunction, and intensifying maladaptive crosstalk between epithelial and immune cells. In fibrotic pathways, persistent epithelial injury activates the NLRP3 inflammasome and drives TGF-β1-mediated fibroblast activation and extracellular matrix deposition, establishing a self-perpetuating cycle of inflammation and remodeling. Emerging data suggest a potential role for MPs in lung carcinogenesis through chronic inflammation, indirect genotoxic effects mediated by oxidative stress, and altered cellular homeostasis. Overall, MPs represent an underrecognized but increasingly relevant environmental factor capable of inducing epithelial damage, promoting chronic airway inflammation, and contributing to the pathophysiology of asthma, COPD, pulmonary fibrosis, and possibly lung cancer. Understanding these mechanisms is crucial to guide preventive strategies, regulatory policies, and future clinical research. This review critically evaluates current experimental evidence on microplastic–epithelium interactions, highlighting mechanistic insights, methodological limitations, and key gaps that must be addressed to clarify their role in airway diseases.

## 1 Introduction

MPs (MPs) and NPs (NPs) pose a significant emerging threat to human health. Due to their widespread presence in air, water, and soil, human exposure is unavoidable, and the respiratory system represents one of the primary interfaces with airborne plastic particles. Increasing experimental and clinical evidence suggests that MPs may have adverse effects on the airways, including inflammatory responses, epithelial alterations, and altered repair responses, underscoring the need for preventive strategies and well-designed clinical studies to define their impact on human health better and to mitigate their effects.

MPs are water-insoluble solid particles or polymer matrices of primary or secondary origin, with regular or irregular shape and dimensions between 1 µm and 5 µm, while particles smaller than 1 µm are called NPs (1). They typically result from the breakdown of plastic objects, tires, synthetic fabrics, and industrial products, and persist in the environment due to their resistance to biodegradation.

The most common polymeric components are polypropylene, polyethylene, polyethylene terephthalate, and polystyrene; other chemical components include plastic additives, organic pollutants, and absorbed heavy metals (2). MPs can also accumulate and release these additives within tissues, causing prolonged exposure to potentially toxic substances. Based on morphology, MPs are classified into five major types: fragments, fibers, foam, pellets, and films (3).

MPs are ubiquitous in the environment: they have been detected in water, air, and even table salt. Importantly, growing evidence demonstrates their presence in human biological samples, including lung tissue, urine, blood, and other biological fluids, raising concerns about their potential systemic and organ-specific effects (1–4).

Once inhaled, particle deposition within the respiratory tract is largely determined by aerodynamic diameter. Larger plastic particles (>5 µm) tend to deposit in the upper airways, whereas particles with smaller aerodynamic diameters can reach distal airways. However, it is important to distinguish between MPs (>1 µm), which predominantly remain confined to the lung and are mainly cleared by alveolar macrophages, and ultrafine NPs (<100–200 nm), which may translocate across the air–blood barrier via transcytosis.

Human exposure to airborne MPs occurs both in occupational settings—such as synthetic textile and plastic manufacturing industries—and in the general population through indoor and outdoor air. Indoor environments represent a major source due to synthetic textiles and household dust, while outdoor air contains MPs derived from traffic-related abrasion, waste incineration, and industrial emissions. Inhalation, therefore, represents a continuous and widespread exposure route.

Experimental studies indicate that MPs and NPs can trigger chronic inflammation, oxidative stress, cell apoptosis, and disruption of the epithelial barrier, mediated by pathways such as TLR2/NF-κB and PI3K-Akt-mTOR, with the consequent release of pro-inflammatory cytokines (e.g., IL-6, IL-8, TNF-α). Experimental studies suggest that exposure may exacerbate respiratory diseases such as asthma and COPD, promote tissue remodeling and fibrosis, and potentially contribute to carcinogenesis (4–9).

This review aims to critically synthesize current experimental and clinical evidence on the effects of inhaled MPs and NPs on the airway epithelium. We focus on how particle size, shape, and surface properties influence epithelial deposition, cellular uptake, inflammatory signaling, oxidative stress, and barrier dysfunction. By integrating mechanistic insights with disease-specific evidence, this review discusses the potential role of MPs in the pathophysiology of asthma, COPD, pulmonary fibrosis, and lung cancer, highlighting key knowledge gaps and priorities for future research.

## 2 MPs and respiratory exposure

MPs can enter the human body via ingestion, inhalation, and dermal contact, causing inflammation, oxidative stress, and potential chronic conditions (10)*.* Once produced or brought into contact with different structures, they can release various chemicals used in their production, including chlorine, phthalates, bisphenols, and brominated flame retardants. Their pathogenic effect is also related to their capacity to absorb various harmful agents, such as heavy metals, organic pollutants, or even allergens, becoming carriers of toxins and environmental triggers (11).

Because MPs and NPs are present in all environments; the exposure includes both primary sources (e.g., synthetic textiles, tire and brake wear, urban and household dust, waste incineration, landfills, and construction) and occupational/industrial sources (e.g., synthetic textile industry, flock industry, and vinyl chloride and polyvinyl chloride industrie s^12^) but also all people in their daily life.

Indoor air is the main source of exposure to MPs due to synthetic textiles (i.e., release of fibers from clothing, carpets, and household furnishings) or household dust (i.e., resuspension of particles from surfaces caused by human activities). Outdoor air includes traffic wear (i.e., rubber and polymer particles resulting from tire and brake abrasion), waste and industry (i.e., release from waste incineration, landfills, and industrial emissions). Recently, even cigarette smoke has been identified as a potential source of microfibers released into the environment.

About the entrance and consequently the effect they may have on the respiratory tract, it is necessary to consider the aerodynamic equivalent diameter (AED), influenced by density and diameter; lower density polymers (e.g., polyethylene) and smaller particles have a higher potential to reach the lower airways (13). AED describes how a particle behaves within the airways, rather than its actual geometric size. It is obtained by adjusting the physical diameter for particle density and shape: denser or more regularly shaped particles have a larger AED, meaning they deposit and penetrate differently within the lung.

For approximately spherical particles:

$$d_{a}\;=\;d_{p}x\sqrt{\frac{\rho_{p}}{\rho_{o}}}$$

For non-spherical particles:

$$\;d_{a}\;=\;d_{e}x\sqrt{\frac{\rho_{p}}{\rho_{o}}}x\frac{1}{\sqrt{\chi }}$$

Where: d_a_ = aerodynamic diameter; d_p_ = physical diameter; $\rho_{p}$ = particle density; $\rho_{o}$ = unit density (1 g/cm^3^); d_e_ = equivalent diameter; χ = dynamic shape factor

As mentioned above, the deposition of particles in the respiratory tract depends on their AED:

**AED of 5–30 µm**: deposit in the upper airways and the nasopharyngeal membranes by inertial impaction.
**AED of 1–5 µm**: deposit in the small airways and the terminal bronchiole by sedimentation and diffusion, where they are removed by alveolar macrophages and lung lymphatics. Particles in the range of 1–2.5 µm usually make their way to the terminal bronchiole, the site of greatest accumulation and tissue destruction, as commonly seen in centrilobular emphysema.
**AED <1 µm**: deposit throughout the respiratory tract and stay airborne longer and gain access to alveoli (13).

The air–blood barrier represents a highly selective interface, and current evidence indicates that only ultrafine nanoparticles, rather than most MPs, are capable of efficiently crossing this barrier (14).

## 3 Airway epithelium: structure, function, vulnerability, and the deposition of MPs

The airway epithelium is a complex structure, made up of different cellular components, which plays a key role in protecting the respiratory system against environmental insults (i.e., pathogens and allergens) and in organizing a mechanical and immune response. It is a multi-layered epithelium formed by specialized cells, such as ciliated cells, goblet cells, club cells, basal cells, neuroendocrine cells, and monocytes, which extends from the nasopharynx to the lower airways (15–17). The epithelium acts as both a physical and immunological barrier that limits contact with potentially harmful external substances and maintains internal homeostasis. Several factors make this dual role possible. A sequence of tight junctions maintains epithelial impermeability, reducing the penetration of inhaled substances into the underlying mucosal layers. Mucus production, on the other hand, traps inhaled particles, preventing them from touching the epithelium, and contains substances as lysozyme, defensins, lactoferrin, and secretory immunoglobulin (Ig), which reduce microbial adhesion and bacterial growth. Mucociliary clearance and the ability to produce cytokines like thymic stromal lymphopoietin (TSLP), interleukin (IL) 33, IL-25, IL-1b, TNF-α, IL-6, and chemokines remove pathogens and activate the inflammatory cascade and airway remodeling (16, 18–20).

For these reasons, the epithelium and its damage, with loss of integrity and function, represent the pathogenetic hub underlying various chronic diseases of the respiratory system. In fact, increased epithelial permeability, greater susceptibility to infections, and altered repair mechanisms caused by chronic exposure to pathogens, allergens, or toxic substances can lead to an uncontrolled inflammatory response, responsible for the onset of diseases such as asthma, COPD, and pulmonary fibrosis (18, 20–24).

The epithelium is vulnerable to MPs and NPs due to a combination of factors related to the particles themselves and the cellular response.

The toxicity and the epithelial damage of MPs and NPs are influenced by size, shape, and surface charge (25). Smaller particles (<1 µm) and NPs can penetrate or be absorbed by the pulmonary epithelium and enter the systemic circulation. The elongated shape of microfibers can make phagocytosis by macrophages incomplete, leading to persistence and continuous release of toxic signals. Surface properties, indeed, affect MPs–cell interactions (26), which determines surface charge (higher values increase cell adhesion and uptake) and surface roughness, which increases physical damage to human cells.

## 4 Mechanisms of microplastic-induced epithelial inflammation and damage

MPs can exert their pathogenic and toxic effects on the airway epithelium through various mechanisms; NPs, penetrating deeper into layers of the mucosa, induce oxidative stress, apoptosis, mitochondrial dysfunction and ferroptosis (27–32) while larger MPs cause mechanical damage, inflammation and alteration of tight junctions, compromising the integrity of the epithelial barrier, promoting pulmonary dysfunction and, in some cases, cellular senescence (33–35) and consequently induction of fibrosis.

The main mechanisms of damage involved are listed below.

### 4.1 Interaction with epithelial cells and endocytosis

The original form of damage that occurs is the interaction between MPs and epithelial cells.

MPs interact with epithelial cells both physically and through cellular internalization mechanisms. First, contact itself can cause damage; larger MPs (>1 µm), particularly irregular or fibrous particles, can cause direct mechanical damage to the epithelium, including abrasion, altered mucus composition, and impaired mucociliary clearance. Studies on respiratory organoids and air-liquid interface (ALI) cultures have shown that microfibers (particularly nylon fibers) and their products can reduce the epithelial regenerative capacity and compromise respiratory organoids formation (6).

Smaller MPs and NPs (<200 nm) are primarily internalized by epithelial cells through endocytic pathways. Multiple endocytic mechanisms are involved, including clathrin-mediated endocytosis, caveolin-mediated endocytosis, and macropinocytosis, with the efficiency of uptake depending on the structural features of the specific MPS (36–39). Long, thin fibers usually undergo incomplete endocytosis, remaining partially in the membrane and causing more prolonged toxic damage over time.

Particle internalization can trigger inflammatory responses and disrupt intracellular organelles, amplifying cytotoxicity. The uptake efficiency also varies among epithelial types: renal and hepatic epithelia exhibit higher endocytic activity, while intestinal cells rely more on macropinocytosis and paracellular diffusion, with greater uptake in proximal vs. distal regions (27).

### 4.2 Oxidative stress, ROS generation, and mitochondrial dysfunction

Various studies have shown that one of the pathogenic mechanisms caused by MPs is an increase in cellular oxidative stress through increased production of oxygen radical species. These mechanisms lead to mitochondrial dysfunction, which contributes to tissue damage and the onset of inflammatory conditions, eventually leading to the development of diseases. The oxidative damage induced by MPs persists over time, leading to a consequent depletion of cellular antioxidant defenses. Once the cell's ability to limit oxidative damage is exceeded, lipid peroxidation (especially of cell membranes), damage to DNA proteins, and consequent activation of inflammatory triggers occur (29, 33, 34). Mitochondria are the primary target of the pathogenic process caused by MPs, which can damage the membranes of these cytoplasmic organelles, compromising ATP energy production and the mechanisms of mitochondrial fusion and fission. Interference with these mechanisms leads to energy dysfunction and subsequent cell death (40, 41). Both oxidative and mitochondrial damage occur in various epithelial tissues (renal, cardiac, and respiratory) and are closely related to the characteristics of the MPs to which they are exposed and the duration of exposure.

Particles of certain MPs, such as polypropylene, polystyrene, and polyethylene, are capable of consuming intracellular glutathione antioxidant reserves, inducing ROS production, and producing inflammatory cytokines. Through activation of NF-κB and related pathways, MPs induce epithelial oxidative stress and cytokine release in bronchial epithelial monocultures; immune cell recruitment, including macrophages and neutrophils, has been demonstrated in *in vivo* models. *In vitro* epithelial models, therefore, capture early pro-inflammatory signaling events rather than cellular immune infiltration. These effects have been demonstrated both in bronchial epithelial cell cultures and in mouse models exposed by intratracheal instillation or inhalation (42–44).

The administration of antioxidants (e.g., NAC, vitamin C) has been shown to mitigate the toxic effects of MPs, highlighting the importance of oxidative damage in the toxicity induced by these substances.

### 4.3 Damage to membrane integrity and cellular response (activation of inflammatory pathways)

**Figure 1:**
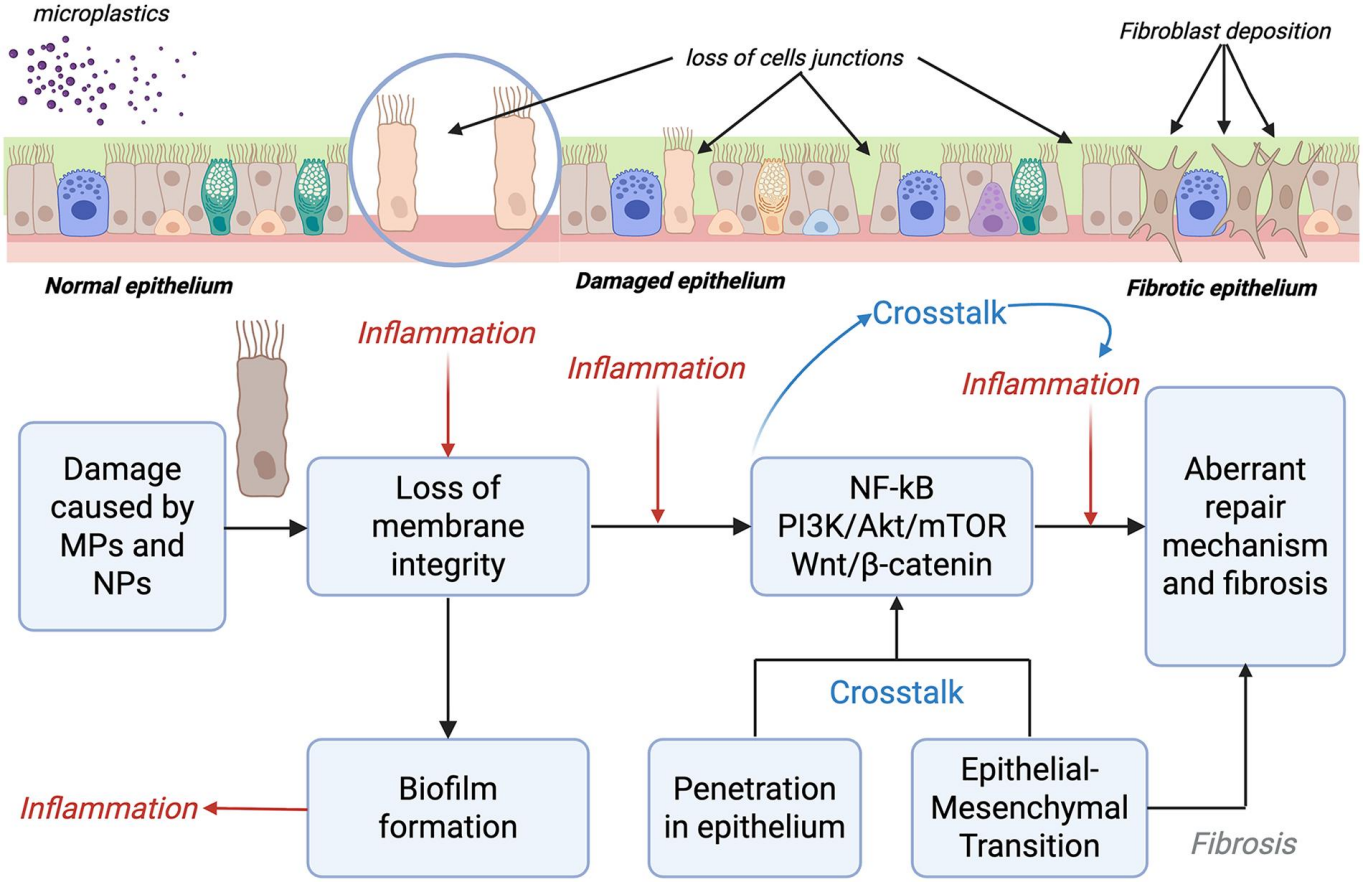
MPs damage on the airway's epithelium.

The loss of membrane integrity is a key damage caused by exposure to MPs, as it represents the starting point for the development of various diseases (particularly in the intestinal and respiratory systems) and the activation of inflammatory pathways. Microplastic exposure has been associated with alterations in junction-related protein expression and activation of inflammatory signaling pathways in epithelial cells; however, direct functional evidence of epithelial barrier disruption remains limited. Increased permeability facilitates the transit of microorganisms and allergens, contributing to inflammatory and hypersensitivity processes that may have implications in the genesis of diseases. Available studies primarily report epithelial stress responses and signaling alterations following microplastic or particle exposure, rather than direct measurements of barrier function (45, 46) (Figure 1).

Signaling pathways such as NF-κB, PI3K/AKT/mTOR, and Wnt/β-catenin—well characterized in epithelial inflammation and cancer biology—have been proposed as potential mediators of microplastic-induced cellular responses, promoting cell damage and tissue changes, although direct microplastic-specific activation in airway epithelium remains to be fully demonstrated (47, 48).

These pathways are indeed known to be interconnected in epithelial and inflammatory biology; activation of PI3K/AKT can inhibit GSK-3β, leading to the accumulation of β-catenin in the nucleus and the activation of the Wnt/β-catenin pathway, which promotes an inflammatory and proliferative response. Oxidative stress, commonly reported following microplastic and nanoplastic exposure, has been shown to activate both PI3K/AKT and NF-κB signalling in various experimental models, potentially amplifying inflammatory responses. In addition, NF-κB activity has been reported to modulate GSK-3β, thereby influencing Wnt/β-catenin signaling.

Experimental evidence supporting activation or modulation of these pathways has been reported primarily in non-respiratory or systemic models. For example, chronic exposure to polystyrene micro- and NPs in mice has been associated with testicular damage and spermatocyte senescence, accompanied by activation of the PI3K/AKT/mTOR pathway. In other studies, exposure to plastic additives, such as DEHP, has shown to induce oxidative stress, necroptosis, and apoptosis in mouse skeletal muscle, with downregulation of the PI3K/AKT/mTOR pathway. These findings highlight the context-dependent nature of pathway modulation following plastic-related exposures and should not be directly extrapolated to the airway epithelium. Inflammatory responses reported in microplastic-related studies are frequently associated with NF-κB activation and increased production of pro-inflammatory cytokines. In addition, an inflammatory state may also be exacerbated by the formation of biofilm around MP or NP surfaces, which can act as carriers for pathogenic microorganisms or concentrate environmental contaminants, thereby potentially enhancing epithelial stress responses (Supplementary Table S1) (47–50).

Emerging evidence also suggests that prolonged exposure to MPs may be associated with epithelial phenotypic changes reminiscent of epithelial–mesenchymal transition (EMT) in selected experimental models. Increased expression of mesenchymal markers such as vimentin and Snail, along with reduced E-cadherin expression, has been reported following long-term exposure to polystyrene or polyethylene particles in animal models, together with extracellular matrix accumulation. However, these observations should be interpreted with caution, as direct functional consequences on epithelial barrier integrity and fibrosis development remain incompletely characterized. EMT-related changes have been proposed to contribute to altered epithelial plasticity and tissue remodeling rather than definitive barrier disruption (51, 52).

### 4.4 Cellular stress and cell death induced by MPs

Further MPs exposure has been reported to induce cellular stress responses in different experimental models, with epithelial cells of the respiratory tract representing the most frequently affected cell types. In bronchial and alveolar cells, exposure to polystyrene MPs has been associated with mitochondrial structural alterations, including mitochondrial shrinkage and fragmentation, dysregulated expression of mitochondrial dynamics regulators (DNM1L/DRP1, MFN2) and impaired energy metabolism. This effect is primarily attributable to oxidative stress, which causes damage to cell membranes, mitochondria, and DNA, inducing apoptosis, autophagy, and ferroptosis. In part, these cellular alterations appear to be associated with inflammatory and oxidative stress–related damage, involving signaling pathways such as PTEN/PI3K/AKT/mTOR and Nrf2-Keap1-HO-1/NQO1, which are known regulators of cellular stress responses and homeostasis. Modulation of these pathways following microplastic exposure has been reported in cellular and limited human observational models, often accompanied by increased expression of cell cycle regulators such as p16 and p21, commonly used as indicators of stress-induced cellular senescence. However, the direct contribution of these pathways to epithelial dysfunction or cell fate decisions remains to be fully elucidated (33, 53–56).

## 5 MPs and respiratory diseases

Chronic inhalation of MPs has emerged as a significant environmental factor capable of triggering and sustaining deep inflammatory and reparative disturbances in the lung, ultimately promoting fibrotic remodeling. Once deposited in the terminal bronchioles and alveoli, particles such as polyethylene, polypropylene, and polyester are recognized as foreign bodies by alveolar macrophages. Their non-biodegradable nature leads to frustrated phagocytosis and persistent activation of innate immune receptors, including TLR2, TLR4, and the NLRP3 inflammasome, initiating NF-κB-dependent transcription of pro-inflammatory mediators and generating high levels of reactive oxygen species (ROS) (57–59). This oxidative and inflammatory microenvironment is a key driver of epithelial injury and chronic immune activation.

### 5.1 Asthma

Evidence from animal models of allergic asthma (treated with dust mites or exposed to ovalbumin) has shown that co-exposure to MPs in these models can aggravate inflammation and allergic characteristics. In fact, in these models, there was an increase in eosinophil recruitment, greater macrophage aggregation, and increased mucus production (60). Other studies have focused on the onset of asthma in individuals chronically and professionally exposed to polyvinyl chloride. Following prolonged exposure, these individuals began to experience asthma-like symptoms, known as “meat-wrapper's asthma”, with references to the occupation that caused such exposure (60). As previously mentioned, MPs and NPs can also act as carriers for pollutants and allergens, as well as pathogenic microorganisms, stimulating epithelial damage and inflammation underlying diseases such as bronchial asthma, allergic rhinitis, and chronic rhinosinusitis, and altering the nasal and pulmonary microbiome (60, 61).

Emerging evidence indicates that inhaled MPs can exacerbate epithelial dysfunction in asthma by disrupting barrier integrity, amplifying oxidative stress, and enhancing maladaptive immune–epithelial interactions. Microplastic particles, including environmentally relevant polyamide fibers, can deposit on the bronchial epithelium and trigger innate immune activation. A recent human *in vitro* study demonstrated that nasal epithelial cells from patients with asthma exhibit a significantly impaired response to microplastic stimulation compared with healthy controls, characterized by reduced epithelial barrier function, altered tight-junction organization, and increased susceptibility to injury (8). When these epithelial cells were co-cultured with monocyte-derived macrophages, microplastic exposure further intensified inflammatory signaling, suggesting that asthmatic epithelium displays an exaggerated and dysregulated interaction with immune cells under microplastic challenge (8). This is consistent with broader toxicological evidence showing that MPs can induce oxidative stress, activate NF-κB signaling, and promote the release of pro-inflammatory cytokines such as IL-1β and IL-18, which are known to aggravate epithelial injury and contribute to chronic airway inflammation in asthma.

Additional mechanistic insights come from studies on human bronchial epithelial cell lines exposed to polyethylene micro- and NPs, which demonstrate induction of epithelial–mesenchymal transition (EMT), downregulation of epithelial markers, and upregulation of mesenchymal proteins, indicating a shift toward a profibrotic phenotype that may contribute to airway remodeling in asthma (4). MPs have also been shown to impair mucociliary function, increase mucus production, and disrupt epithelial repair processes, all of which are central to asthma pathophysiology. Systematic reviews of microplastic inhalation further highlight their capacity to accumulate in the airways, induce oxidative damage, and promote inflammatory responses that disproportionately affect individuals with pre-existing respiratory diseases.

MPs and NPs have demonstrated, in mouse models and *in vitro*, their ability to interact and cooperate with allergens or environmental pollutants, thereby amplifying cellular damage. As regards interaction with allergens, MPs, thanks to their hydrophobic surface, can absorb allergens (especially mites and pollen), leading to the formation of “protein crowns” and conveying a greater number of allergens to the airways. In addition to their role as carriers, MPs can cause structural alterations to the allergen. One example is polystyrene, which can modify the conformation of the main mite allergen (Der p 1), increasing its aggregation and ability to bind IgE, thereby enhancing its allergenicity. This effect is size-dependent, i.e., it is greater when the MPs are smaller (60, 62). In addition to these effects, MPs can increase damage to the epithelium, increasing its permeability and allowing the passage of a greater number of allergens. They are also capable of amplifying the Th2 response. In fact, in mouse models with allergens (HDM, ovalbumin), co-exposure to PM increases eosinophils, IgE, IL-4/IL-5/IL-13, IL-33, and mucus production, aggravating allergic asthma (5, 60, 63). Even in the case of environmental pollutants, MP can act as carriers, particularly for lipophilic additives (phthalates, bisphenol A, PFAS, pesticides). Once these reach the lungs, they can contribute to inflammation, redox imbalance, and the development/exacerbation of asthma. In the case of plasticizers such as DEPH, however, in a mouse model of allergic asthma (OVA), inflammation, bronchial hyperreactivity, oxidative stress, and Th2 response are significantly worse than with PS-MPs or DEHP alone, via activation of the TRPA1-p38 MAPK pathway (60, 64). MPs, part of the PM2.5/PM10 fraction, can adsorb PAHs, PCBs, pesticides, and microorganisms, with possible local release and additive or synergistic effects on inflammation and oxidative stress in the airways (65).

Collectively, current evidence suggests that microplastic exposure can worsen bronchial epithelial fragility in asthma by compromising barrier integrity, enhancing oxidative and inflammatory stress, and promoting structural changes that may accelerate airway remodeling.

### 5.2 COPD

There are also several correlations between MPs and NPs and chronic obstructive pulmonary disease (COPD). Some *in vitro* studies have shown that exposure to polystyrene microparticles in bronchial epithelial cell lines can reduce the expression of alpha-1 antitrypsin, the reduction of which can lead to the development of emphysema. Plastic additives, such as bisphenol A, have also been linked to the incidence of COPD in epidemiological studies. Cigarette smoke, a key element in the pathogenesis of COPD, contains various types of MPs ranging in size from 20 to 500 microns. Analysis of the bronchoalveolar fluid of smokers, compared to non-smokers, has shown the presence of large amounts of MPs of various types in the former, suggesting a possible role for these in the pathogenesis of COPD (60). Traditional cigarettes contain a cellulose acetate filter that continuously releases MPs. This release causes chronic exposure in these patients and contributes to the pathogenesis of COPD. Other MPs found in greater numbers in smokers than in healthy individuals are polyurethane and silicone. These substances are irritants to the mucous membranes, and chronic exposure to them can lead to persistent inflammation of the airways, a key factor in bronchial remodeling and progressive airflow limitation. This inflammatory response is central to the pathophysiology of COPD (66, 67). It has also been demonstrated that exposure to these substances has an additive or synergistic effect with cigarette smoke on the loss of respiratory function. This is consistent with the pathophysiology of COPD, in which persistent inflammatory stimuli lead to remodeling and reduction of FEV₁ (68).

Human and animal studies together indicate that inhaled MPs can exacerbate epithelial injury in COPD through converging inflammatory and oxidative mechanisms, although the type of evidence differs substantially between experimental systems. Human data come primarily from *in vitro* exposure studies using epithelial cells derived from COPD patients: nasal epithelial cells exposed to polyamide microplastic fibers show impaired barrier integrity, disrupted tight-junction organization, and heightened susceptibility to injury, effects that are further amplified when cells are co-cultured with macrophages, indicating an abnormal epithelial–immune interaction specific to COPD (69). Additional human observational evidence confirms that MPs can accumulate in lung tissue and are associated with inflammatory signatures, supporting the biological plausibility of epithelial involvement *in vivo* (60). In contrast, animal studies provide mechanistic confirmation of downstream injury pathways: rodent models exposed to MPs develop epithelial thickening, oxidative stress, NF-κB and NLRP3 activation, and increased expression of TNF-α and IL-1β, changes that precede fibroblast activation and early fibrotic remodeling (34, 70). These findings align with broader toxicological evidence showing that micro- and NPs induce oxidative stress and inflammation in lung tissue across species (34). Integrating these results, human studies demonstrate that COPD epithelium is intrinsically more vulnerable to microplastic-induced dysfunction, while animal models confirm that such exposure can initiate the full cascade of epithelial injury, inflammasome activation, and profibrotic signaling. Together, they support a coherent model in which MPs worsen epithelial fragility in COPD and may accelerate airway remodeling through combined barrier disruption, oxidative stress, and dysregulated macrophage–epithelium crosstalk.

### 5.3 Fibrosis

More solid evidence comes from the analysis of the long-term effects of occupational exposure. Chronic exposure to plastics, such as PVC and synthetic fibers (nylon and polypropylene), is associated with an increased risk of pulmonary fibrosis and pneumoconiosis. The mechanism responsible for this development is frustrated phagocytosis (a mechanism shared with other pneumoconioses), in which macrophages are unable to destroy particles that are too large or difficult to eliminate, leading to chronic production of inflammatory mediators with an aberrant repair mechanism that causes collagen deposition and, consequently, fibrosis (22, 44, 60). Activated macrophages release cytokines such as TNF-α, IL-1β, IL-6, and IL-18, which recruit neutrophils and monocytes and perpetuate a non-resolving inflammatory state (57, 58, 69). NOD-, LRR- and pyrin domain-containing protein 3 (NLRP3) inflammasome activation, repeatedly observed in experimental models exposed to MPs, promotes the maturation of IL-1β and IL-18, amplifying epithelial damage and reinforcing the cycle of injury and repair (58, 69, 71, 72). Persistent injury to type I and type II alveolar epithelial cells impairs normal regenerative capacity and stimulates the release of Transforming Growth Factor beta 1 (TGF-β1), the central cytokine orchestrating pulmonary fibrogenesis (58, 69, 72). TGF-β1 activates both canonical Small mother against decapentaplegic (Smad2/3) signaling and non-canonical pathways, such as Mitogen-Activated Protein Kinase (MAPK) and osfoInositide-3-Chinasi/Proteina Chinasi B (PI3K/Akt), driving epithelial–mesenchymal transition, fibroblast activation, and differentiation into α-SMA-positive myofibroblasts. These cells synthesize excessive extracellular matrix components, including collagen types I and III, progressively replacing normal alveolar architecture. In parallel, microplastic-induced inflammation enhances the expression of chemokines such as CCL2/MCP-1, which recruit circulating monocytes and promote their polarization toward an M2 macrophage phenotype. M2 macrophages secrete TGF-β1, IL-10, and arginase-1, reinforcing fibroblast survival and sustaining extracellular matrix deposition. Oxidative stress generated directly by microplastic surfaces or indirectly through cellular activation further damages epithelial lipids, proteins, and DNA. This oxidative milieu maintains NF-κB and AP-1 activation and promotes myofibroblast persistence through STAT3 signaling, consolidating the fibrotic response (58, 59). The convergence of epithelial injury, chronic inflammation, macrophage polarization, and fibroblast activation establishes a self-perpetuating loop in which each process amplifies the next, ultimately leading to irreversible parenchymal remodeling. Smoking, moreover, increases the entry and retention of MPs in the lungs; through chronic inflammation, oxidative stress, ferroptosis, and activation of profibrotic and pro-tumor pathways, these MPs could amplify the tendency toward fibrosis, representing a co-factor with respect to MPs capable of inducing this type of lung damage (60, 67).

Overall, microplastic-induced lung injury arises from a tightly interconnected network of innate immune activation, oxidative stress, cytokine release, inflammasome signaling, and profibrotic pathways. These mechanisms transform chronic inflammation into progressive fibrosis, compromising alveolar structure and respiratory function.

### 5.4 Lung cancer

The most worrying aspect in terms of global health is undoubtedly the development of lung cancer. This increased risk is the result of a combination of the pathogenic mechanisms described above. Chronic exposure to these plastics causes chronic inflammation, which is responsible for remodeling the structure of the airways, aberrant repair mechanisms, and increased oxidative stress, leading to DNA damage with potential neoplastic evolution. Some MPs, in fact, such as PVC in both monomers and powder form, have been linked to an increased risk of developing lung cancer (60). Growing evidence suggests that inhaled MPs (MPs) and NPs (NPs) may contribute to lung carcinogenesis through a combination of physical persistence, chemical toxicity, and chronic inflammatory signaling. MPs have been detected in human lung tissue, confirming that airborne particles can deposit deeply in the respiratory tract and remain for prolonged periods, especially in distal airways where clearance mechanisms are limited (73). Microplastic fibers were found at approximately twice the density in lung tumors compared to adjacent healthy tissue, suggesting bioaccumulation in the neoplastic microenvironment. Their surfaces often carry additives or adsorbed pollutants, potentially amplifying biological effects. These additives also include carcinogenic contaminants such as phthalates, BPA, and PAHs, and this co-exposure can enhance the carcinogenic effect of smoke on lung tissue (74, 75). Furthermore, *in vitro* co-exposure to PET, NPs and cigarette smoke condensate synergistically increases ROS, DNA damage, neoplastic transformation, migration, and invasiveness of BEAS-2B bronchial cells, with downregulation of tumor suppressor genes (76).

Experimental studies increasingly support a mechanistic connection between MP exposure and malignant transformation. Researchers at MedUni Vienna recently demonstrated that MPs could induce malignant changes in human lung cells, including alterations associated with cancer development, highlighting direct carcinogenic potential *in vitro* (77). These findings align with broader toxicological literature showing that MPs trigger oxidative stress, mitochondrial dysfunction, DNA damage, and activation of pro-inflammatory pathways such as NF-κB and MAPK—processes central to tumor initiation and promotion. A comprehensive review published in Cancers synthesizes these mechanisms, emphasizing how MPs may influence both the risk and progression of lung cancer by modulating cellular pathways, promoting chronic inflammation, and potentially affecting therapeutic responses (73). Another review from UCSF underscores that airborne MPs behave similarly to particulate air pollution, a well-established carcinogen, and may contribute to chronic pulmonary inflammation that increases lung cancer risk (78). Despite these mechanistic insights, epidemiological evidence remains limited. Current data are largely extrapolated from toxicological studies and from the known health impacts of particulate pollution. Nonetheless, the rapid global rise in environmental microplastic contamination has intensified concerns, prompting calls for longitudinal studies and stronger regulatory measures. Overall, while causality is not yet established, converging evidence from cellular, molecular, and environmental research suggests that MPs represent a plausible and increasingly urgent risk factor in lung cancer biology.

**Figure 2:**
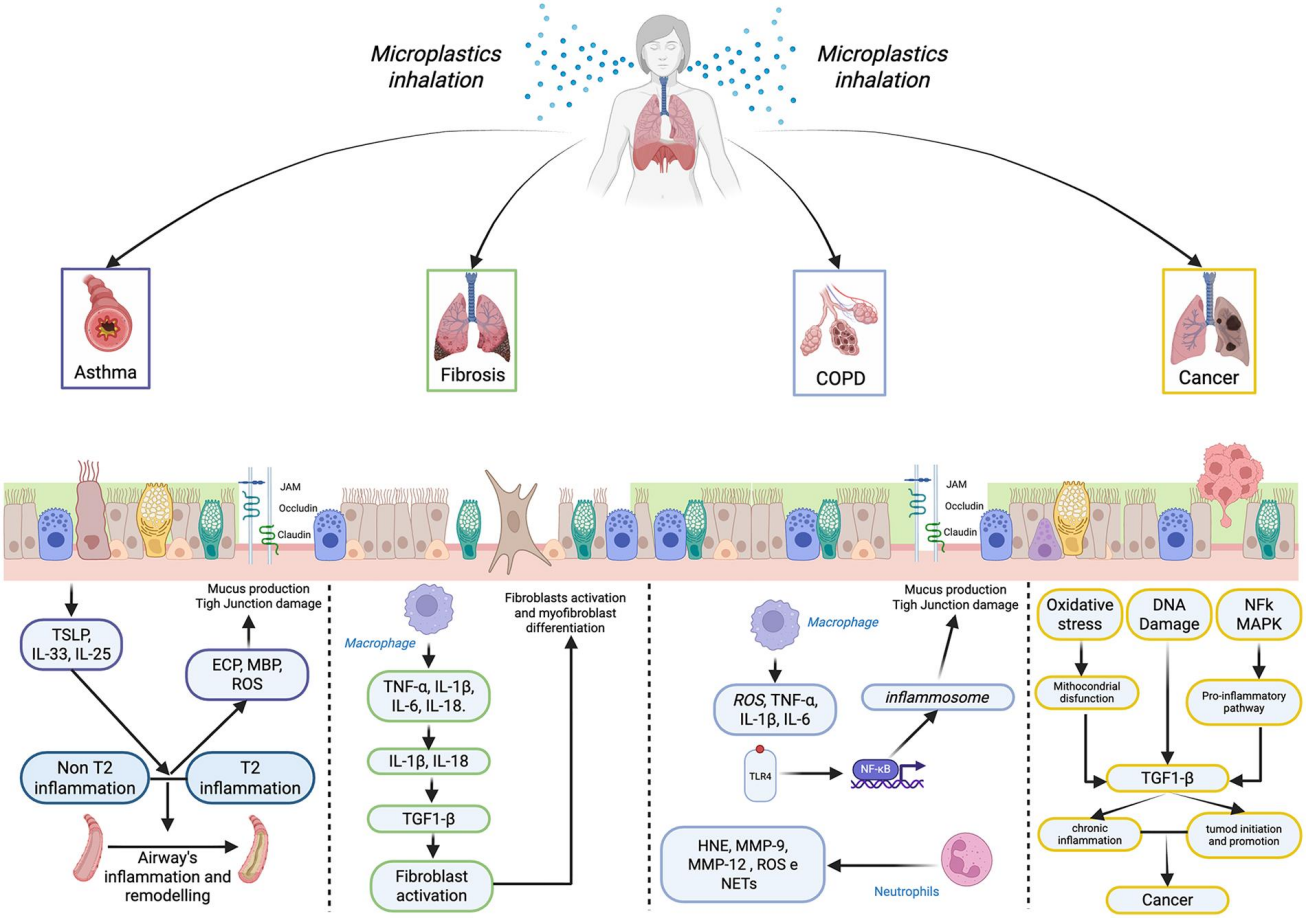
MPs and NPs damage mechanisms in asthma, COPD, fibrosis, and lung cancer. TSLP, thymic stromal lymphopoietin; IL, interleukin; ECP, cationic eosinophil protein; ROS, reactive oxygen species; MBP, major basic protein; T2, type 2; non T2, non-type 2; JAM, junctional adhesion molecules; TNF-α, tumor necrosis factor alpha; TGF1-β, transforming growing factor 1; TLR-4, toll-like receptor; HNE, human neutrophils elastase; MM-9, matrix metalloproteinase-9; NET, neutrophil extracellular traps; NF-kB, nuclear factor kappa-light-chain-enhancer of activated B cells; MAPK, mitogen-activated protein kinase.

Further, more in-depth studies are certainly needed to explain the role of MPs in the pathogenesis of chronic airway diseases (asthma and COPD) and in determining their exacerbations, as well as their potential but real carcinogenic risk (Figure 2).

## 6 Gaps, limitations, future directions

Notwithstanding the growing body of evidence confirming the presence of micro- and NPs (MNPs) in human lung tissue and their biological capacity to induce inflammation and potential neoplastic damage, science still faces crucial methodological challenges. The strength of the information we have lies precisely in our ability to clearly outline what is missing to establish a definitive causal link between daily environmental exposure to MNPs and respiratory diseases.

One of the weaknesses of current research is the difference between environmental conditions and real-world conditions. Most *in vitro* and animal model research is conducted using excessively high doses, often orders of magnitude higher than those normally inhaled by an individual. Furthermore, the particles tested are predominantly spherical and in their original condition, chemically pure, such as polystyrene. This approach, while useful for revealing basic toxicity mechanisms, does not reflect the environmental reality, where MNPs are irregular in shape (fibers, fragments) and chemically altered (with additives and absorbed pollutants). The second gap is the lack of rigorous environmental data. We need a much more accurate assessment of the actual levels of exposure to MNPs that we face daily, both outdoors and indoors. It is imperative to know exactly how many particles of what size and chemical composition reach our lungs to compare this data with the doses used in the laboratory.

Finally, the characterization of particles inside the human body represents a significant technical hurdle. We urgently need to develop improved analytical techniques to detect and identify MNPs in biological samples, not only in diseased lungs, but also in healthy ones. Only through detailed analysis of the size, polymer type, and shape of deposited MNPs will we be able to definitively correlate a specific type of plastic pollutant with the onset or progression of diseases such as asthma, COPD, or lung cancer.
