# Supplementary material for: Silent invaders: the role of MPs on epithelium inflammation and damage in airway diseases
Source: Front Allergy. 2026 Feb 16;7:1758940. doi: 10.3389/falgy.2026.1758940 (PMC12950594; doi:10.3389/falgy.2026.1758940)
Supplement: Supplementary file 1 [file Table1.docx]

**Table 1. Pathogenic mechanisms associated with microplastic exposure.**

| Process Step | Mechanistic Description | Key Molecules/Markers | Pathological Consequences |
| --- | --- | --- | --- |
| Microplastic exposure | Initial contact with MPs/NPs triggers cellular stress | — | Initiation of damage pathways |
| Loss of membrane integrity | Disruption of tight junctions and adhesion complexes | Occludins, ZO-1, E-cadherin | Barrier dysfunction |
| Increased epithelial permeability | Facilitates translocation of particles, allergens, pathogens | — | Enhanced susceptibility to inflammation |
| Penetration into epithelium | MPs/NPs infiltrate tissue layers | — | Activation of intracellular signaling cascades |
| Inflammatory pathway activation | Induction of pro-inflammatory signaling | NF-κB, PI3K/Akt/mTOR, Wnt/β-catenin | Cytokine release, oxidative stress |
| Pathway crosstalk | Synergistic amplification via GSK-3β inhibition and β-catenin accumulation | GSK-3β, β-catenin | Tissue remodeling, proliferation |
| Oxidative stress and cell death | ROS-mediated damage, apoptosis, necroptosis | ROS, caspases, necroptosis markers | Tissue injury |
| Biofilm formation | MPs/NPs act as substrates for biofilm harboring pathogens/toxins | — | Persistent inflammation |
| Epithelial–mesenchymal transition (EMT) | Phenotypic shift promoting migration and fibrosis | ↑ Vimentin, Snail; ↓ E-cadherin | Loss of barrier integrity, fibrosis initiation |
| Aberrant repair and collagen deposition | Excessive ECM production and remodeling | Collagen, fibronectin | Fibrosis-like patterns |
| Chronic inflammation and fibrosis | Long-term pathological state | — | Disease progression |
